# How Useful Is Endoscopic Ultrasound in Differentiating T3/T4a T Stage of Colorectal Cancer: A Prospective Study

**DOI:** 10.3389/fonc.2021.618512

**Published:** 2022-01-21

**Authors:** Chaoqun Han, Xuelian Tang, Ming Yang, Kun Zhang, Jun Liu, Rong Lin, Zhen Ding

**Affiliations:** ^1^ Division of Gastroenterology, Union Hospital, Tongji Medical College, Huazhong University of Science and Technology, Wuhan, China; ^2^ Division of Pathology, Union Hospital, Tongji Medical College, Huazhong University of Science and Technology, Wuhan, China

**Keywords:** endoscopic ultrasound, colorectal, seminal vesicle, cervix, staging, accuracy

## Abstract

**Objective:**

Endoscopic ultrasound (EUS) is an established method for staging of colorectal cancer. Nevertheless, prior assessments of its T stage accuracy have been limited, particularly ambiguity in assessed T3 and T4a stage. This study was to characterize the EUS image features and pay attention to distinguish T3 from T4a T stage.

**Methods:**

A total of 638 patients who prospectively underwent colorectal EUS were recorded. The final diagnoses were compared with the concurrent or follow-up histopathology. Univariate and multivariate logistic regressions were used to assess variation in diagnostic performance with case attributes.

**Results:**

The accuracies of EUS in classifying colorectal cancer for overall, T1, T2, T3, and T4a stages are 73.04, 62.32, 67.46, 71.26, and 83.52%, respectively. With attention to EUS image features, the lesion penetrates the entire wall and locates below the seminal vesicles or cervix is T3 stage. If the lesion locates above clearly-defined space between the anterior rectal wall and the posterior surface of the seminal vesicles or cervix, we identify as T4a stage; However, when located above seminal vesicles or cervix but on the posterior wall of the rectum, the lesion still considers as T3 stage. The tumor location and histological type are associated with inaccuracy T stage.

**Conclusions:**

EUS provides reliable diagnostic accuracy in the colorectal cancer stage. The seminal vesicles and cervix are the important markers to predict infiltration depth for T3/T4a stage. Furthermore, the tumor location, histological type, and EUS image features for each tumor T stage should warrant attention.

## Introduction

Accurate categorization of locoregional rectal cancer staging is crucial for prognostic assessment and making initial therapeutic decisions for patients. Endoscopic ultrasonography (EUS) provides detailed images and has emerged as an integral part of the staging of rectal cancer. In many studies involving colorectal cancer, the accuracy of EUS for T staging ranged from 70 to 93%, compared with 65 to 75% for computed tomography, and 75 to 85% for magnetic resonance imaging. EUS is indeed considered as the first-choice imaging modality for regional staging of colorectal cancer compared to other methods (1–6).

However, according to the recent edition of the cancer staging handbook of the American Joint Committee on Cancer (AJCC 8th) dated 2017 or AJCC 7th published 2010, the T3 lesions have been defined as an invasion into the subserosa or into the colorectal fat tissue(no visceral peritoneum covering); while the T4a stage penetrated through the peritoneum(serosa) but not having invaded an adjacent organ (7, 8). As we all know, from a technical perspective, distinguishing between subserosal and serosal lesions by EUS is challenging. EUS also fails to detect peritoneal reflection and ligaments. Therefore, T3/T4a stage encompasses the greatest variance than any other T category. Prior articles showed a high accuracy rate of EUS in the preoperative staging of rectal cancer, However, none discussed how they distinguished T3 from T4a when their got the accuracy in each stage. The concept of T3 and T4a are used indistinctly in the EUS workup.

The peritoneal reflection is a thin, translucent, serous membrane and is the most complexly arranged serous membrane in the body (9). Rectovesical ponch (rectovesical peritoneal reflection) is identified as the lowest peritoneum in the male pelvic cavity which is a transverse view at the level of the seminal vesicles. Douglas’ Pouch is the lowest peritoneum in the female pelvic cavity which is a transverse view at the level of the cervix (10, 11). EUS provides the accurate ability to differentiate anatomic structural of seminal vesicles or cervix and show remarkable features in their echogenic appearance. So, could seminal vesicles or cervix be used as markers for distinguishing T3 fromT4a stage? The original aim of the study is to prospectively assess the preoperative staging accuracy with a focus on T3/T4a by endorectal EUS according to seminal vesicles or cervix EUS features. Furthermore, previous studies have not reported the factors influencing the T stage of colorectal cancer, so we also attempted to identify sonographic features that affect the accuracy of EUS T staging.

## Methods

### Patient Selection

A total of 936 patients with colorectal cancer over the 3-year study period (September 2016 to September 2019) were included. All patients met the following criteria: (1) their diseases were pathologically proved colorectal cancer through a colonoscopy; (2) they underwent tumor-free resection (R0) margin status; and (3) they underwent pretreatment EUS. The exclusion criteria were: (1) any exposure to chemotherapy or radiation before EUS; (2) patients undergoing emergency surgery or palliative treatment; (3) patients with multiple malignancies or previous other primary cancers; (4) tumors with obstruction that EUS failed to pass through. In such tumors placing the transducer perpendicular to the tumor is difficult, rendering them more likely to be misstaged; and (5) lack of available surgical pathology data. The study was approved by the Ethics Committee of Tongji Medical College, Huazhong University of Science and Technology (IORG No: IORG0003571). All patients provided written informed consent for EUS operation and their information had been anonymized and de-identified.

### EUS Equipment and Technique Procedures

Curved heteroscope with a 360-degree radial echoendoscope (Olympus processor EU-ME2, Olympus, Tokyo, Japan) or forward viewing radial echoendoscope (EG-530UR2 and an Ultrasound Processor SU-9000 (FUJIFILM Corporation, Tokyo, Japan) were used, depending on localization of the tumor. If the lesion was located in the rectum, we used the curved heteroscope. When the tumor was beyond the rectum sigmoid junction colon, we always used with the latter one (FUJIFILM), which has a front endoscopic view, and the scope could be easily passed into the right side of the colon. We usually used 7.5 MHz for the staging of the tumor, as it was considered the optimal frequency to provide the best endosonographic imaging; however, the frequency was adjusted from 6 to 12 MHz to acquire the finest image. After preparation by rectal enema and inspection, the rectal lumen was filled with de-aerated water to assist acoustic coupling and to provide optimal EUS visualization. All operations were performed by three experienced gastroenterologists with a track record of more than 1,000 EUS per year.

### Methods for Evaluation of Colorectal Cancer T Staging by EUS

**Figure 1:**
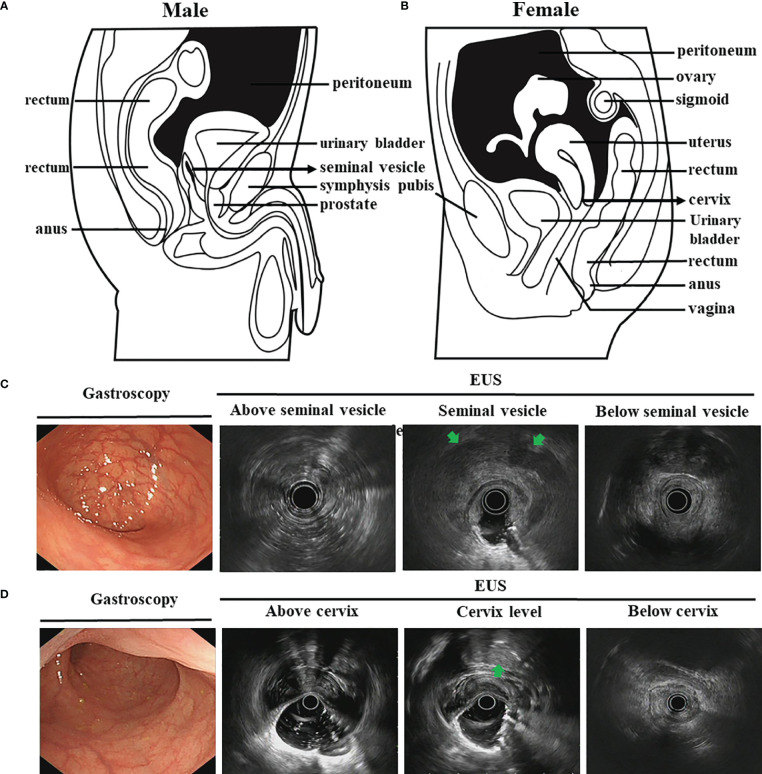
The schematic diagrams and EUS images for normal pelvic viscera and peritoneal reflection. **(A, B)**. The schematic diagrams show normal pelvic viscera and peritoneal reflection for male **(A)** and female **(B)** (black shaded area); **(C, D)**. EUS images for normal pelvic viscera about white light endoscopy, peritoneal reflection marker for male seminal vesicle **(C)** and female cervix level **(D)**. The seminal vesicles and cervix are shown at the arrowheads.

Tumor stage was evaluated according to the new AJCC 8th TNM staging system. Analogous to pathologic classification, the extent of wall invasion was imaged as a hypoechoic disruption and evaluated based on the tumor infiltration into each layer (**Table 1**) (12, 13). For this prospective study, patients were included if the distal margin of the tumor was within 15 cm from the anal verge (14). In order to assess EUS accuracy: (1) We first used previously published method (15, 16), dividing tumor site into low, middle, or upper based on the distance of the tumor margin to the anus: upper third from 12 to 15 cm, middle third from 8 to 12 cm, and lower third from 7 cm to the anus; (2) Then, we used the new evaluation criterion. With special attention to the location of seminal vesicles and tumor during EUS: for T1 and T2 stage, the method is the same as published. For T3 stage, images show the lesion invades throughout the entire wall and locates below the seminal vesicles or cervix. When located above seminal vesicles or cervix but on the posterior wall of the rectum, the lesion is still considered as a T3 stage. However, if the lesion locates above clearly-defined space between the anterior rectal wall and the posterior surface of the seminal vesicles or cervix, we identify it as a T4a stage (**Table 1** and **Figure 1**).

**Table 1:** The EUS features and AJCC 7th/8th staging system for primary colorectal cancer.

Primary tumor (T)	Ustage^1^	Our criteria	P stage^2^
**T1**	Tumor localized in the mucosa (T1a) or submucosa (T1b) and do not extend beyond the first three echo layers	EUS images show disappearance, mild thickness and hypoechoic change of the first two hypoechoic layer, and normal (T1a) or interrupt (T1b) third hyperechoic layer	T1a: Intraepithelial or invasion of lamina propria T1b: Tumor invades submucosa
**T2**	Tumor has infiltrated the muscularis propria, but is localized in the rectal wall, with some destruction visible, and thickened low echoes in the muscle layer	EUS images of the lesion show disappearance of the first three layers and companied by muscularis propria visible indistinctly or obvious thickening	Tumor invades muscularis propria
**T3**	Tumor invades through the muscularis completely and may even extend beyond the five echo layers into the perirectal space	EUS images show the lesion invades throughout the entire wall and locates below the seminal vesicles and cervix or locates at the posterior rectal wall but above seminal vesicles and cervix	Tumor penetrating beyond the muscularis propria, invading the subserosa or arriving at colorectal fat tissue (no visceral peritoneum covering)
**T4a/T4b**	T4a: Tumor invades the visceral peritoneum with irregular low echo jagged protrusions which are suggestive of tumor involvement of tissue outside of the intestinal wall T4b: tumor involvement of adjacent organs or tissues (prostate or vagina, etc.)	EUS images show the lesion invades throughout the entire wall and locates above clearly-defined space between the anterior rectal wall and the posterior surface of the seminal vesicles and cervix (T4a) or tumor involvement of adjacent organs or tissues (prostate or vagina, etc.)	T4a: Tumor having perforated the visceral peritoneum(serosa) but not having invaded an adjacent organ; T4b: Tumor penetrating the adjacent organ.

^1^ustage was T staging definition of colorectal carcinoma by EUS.

^2^pstage was T staging definition of colorectal carcinoma by pathology.

### Data Collection

In order to identify features that affect the accuracy of EUS T staging, we also focused on the case attributes of interest, namely, demographic information (patient age, sex), histological details pertaining to colorectal diagnosis (distance of the tumor from the anal verge, stage and histological type), white light endoscopy, and ultrasonic characteristics (visualized tumor size, ascites, the percent of circumferential involvement, detailed location of the lesion, orientation, the relationship between lesion and seminal vesicles or cervix). A challenge in the identification of nodes with EUS is the inability to visualize nodes that are outside the range of the transducer. Thus, rectal cancer N staging remains to be an area of uncertainty (17). The efficacy of EUS N staging and other related data are not shown.

### Statistical Analysis

Categorical variable results are presented as numbers and percentages, and continuous variables are presented as mean ± standard deviation (SD). The possible influence of variously categorical or non-categorical variance was conducted by Pearson’s chi-squared test and *t*-tests. Subsequently, logistic regression models were performed to assess potential associations relate to EUS accuracy. Statistical analysis was performed using IBM SPSS Statistics software (version 20.0, IBM Corp, Armonk, NY, USA). A significance level of *P* ≤0.05 was used for all models (two-sided).

## Results

### General Patient Characteristics

In total, 638 (mean age, 57 y; range, 25–85 y) cases undergoing EUS stage were prospectively enrolled and met the inclusion criteria. Approximately 62.1% of the patients were male. The main presenting symptoms were bowel habit changes (13.4%), hematochezia (79.2%), melena (9.5%), abdominal pain (35.8%), anemic symptoms (25%), weight loss (12%), and partial gut stricture (n = 4.7%). Measured tumor size ranged from 0.8 to 6.5 cm (median, 2.85 cm), and mean distance of the tumor from the anal verge was 8.8 cm (range 3–60 cm). With consideration to the location of lesions, 296 tumors (46.39%) were located above the peritoneal reflection and T3 cases accounted for the majority of cases. For colon cancer, the lesions were located at the cecum (n = 9), ascending colon (n = 16), transverse colon (n = 21), descending colon (n = 35), and sigmoid colon (n = 59). Tumors were well differentiated in 10.0%, moderately differentiated in 63.5%, poorly differentiated in 18.8%, and signet ring cell adenocarcinoma in 7.7%, respectively. We also focused on EUS image characteristics, namely, presence of circumferential lesions (cancer extension beyond a semi-circular area, 41.5% of tumors were circumferential lesions≥1/2), and ascites (4.7%). The demographic and clinical characteristics are summarized in **Table 2**.

**Table 2:** The Basic clinicopathological characteristics of the patients and tumors.

Characteristic	No. of patients (%)
Age (year)	
Mean ± SD (rang)	57.0 ± 10.8 (25–85)
Median (P25, P75)	58.0 (45.0, 65.0)
Gender	
Male	396 (62.1%)
Female	242 (37.9%)
Tumor location	
Rectum	498 (78.1%)
Colon	140 (21.9%)
Distance, cm, ±SD (range) from the anal verge to the distal border of the tumor^*^	8.8 ± 4 (3–60)
Location in relation to peritoneal reflection, no. (%) †	
Below	342 (53.61%)
Above	296 (46.39%)
Tumor located at rectum	
Upper third	115 (23.09%)
Middle third	309 (62.05%)
Lower third	74 (14.86%)
Cross-sectional portions	
Circumferential lesions ≥1/2	265 (41.5%)
Circumferential lesions <1/2	373 (58.5%)
Ascites^†^	30 (4.7%)
Absence of ascites†	608 (95.3%)
Histological type	
Well-differentiated	64 (10.0%)
Moderately differentiated	405 (63.5%)
Poorly differentiated	120 (18.8%)
Signet ring cell adenocarcinoma	49 (7.7%)
8^th^ AJCC pathologic T category	
pT1	69 (10.82%)
pT2	126 (19.75%)
pT3	261 (40.91%)
pT4	182 (28.52%)

SD, standard deviation; AJCC, American Joint Committee on Cancer; pT, pathological T stage; For histological type, a patient may have two, such as moderately and poorly differentiated types, the worse was for the final result.

^*^Data based on EUS.

^†^Data based on pathology.

### Efficacy of EUS in Classifying Colorectal T Stage

Compared with pT category, the overall accuracy of EUS in classifying colorectal T category was 73.04% and overstaging (15.67%) was more common than understaging (11.29%). With regard to T1 cases, our data showed that EUS had unsatisfactory accuracy and high overstaging rates (37.68%). Only 62.32% of pT1 patients were actually diagnosis and 78.19% (43/55) of uT1 patients were actually pT1 cases. In pT2 cases, 67.46% were accurately classified, but as many as 23.02% was overstaged as uT3 or uT4 lesions by EUS.

EUS had the highest accuracy (83.52%) in pT4a colorectal patients. However, note that as many as 17.24% of pT3 patients were overstaged as having uT4 lesions by EUS. Approximately 11.49% of pT3 patients identified by EUS were understaged from uT2 cases. The majority (30/182 cases) of understaging occurred in patients with EUS T4 tumors, eventually found to have pathological pT3 (24 cases) and pT2 (6 cases), as assessed by the resected specimens. We also separated the results of rectal and colon cancer T stage respectively. Detailed comparisons between uT and pT categories for all colorectal, rectal and colon cancer patients are presented in **Table 3**.

**Table 3:** Results of endosonography (uT) categories and pathologic T (pT) categories for (1) all patients, (2) rectal cancer patients, and (3) colon cancer patients.

(1) pT categories	uT categories (AJCC 8th)							
		T1	T2	T3	T4	Accuracy, %	Overstage, %	Understage, %
T1	69	43	18	8	0	62.32	37.68	0
T2	126	12	85	21	8	67.46	9.52	23.02
T3	261	0	30	186	45	71.26	17.24	11.49
T4	182	0	6	24	152	83.52	0	16.48
pTtotal	638	55	139	239	205	73.04	15.67	11.29
(2) pT categories	uT categories (AJCC 8th)							
		T1	T2	T3	T4	Accuracy, %	Overstage, %	Understage, %
T1	69	43	18	8	0	62.32	37.68	0
T2	111	11	74	18	8	66.67	9.91	23.42
T3	202	0	21	150	31	74.26	10.40	15.34
T4	116	0	6	19	91	78.45	0	21.55
pTtotal	498	54	119	195	130	71.89	16.67	11.44
(3) pT categories	uT categories (AJCC 8th)							
		T1	T2	T3	T4	Accuracy, %	Overstage, %	Understage, %
T1								
T2	15	1	11	3	0	73.34	6.66	20.00
T3	59	0	9	36	14	61.02	15.25	23.73
T4	66	0	0	5	61	92.42	0	7.58
pTtotal	140	1	20	44	75	77.14	12.14	10.72

p, pathological; u, ultrasonographic; AJCC American Joint Committee on Cancer. Because we didn't diagnose T1 stage of colon cancer, it is expressed as “–”.

### The EUS Image Features for Different Tumor T Stages

**Figure 2:**
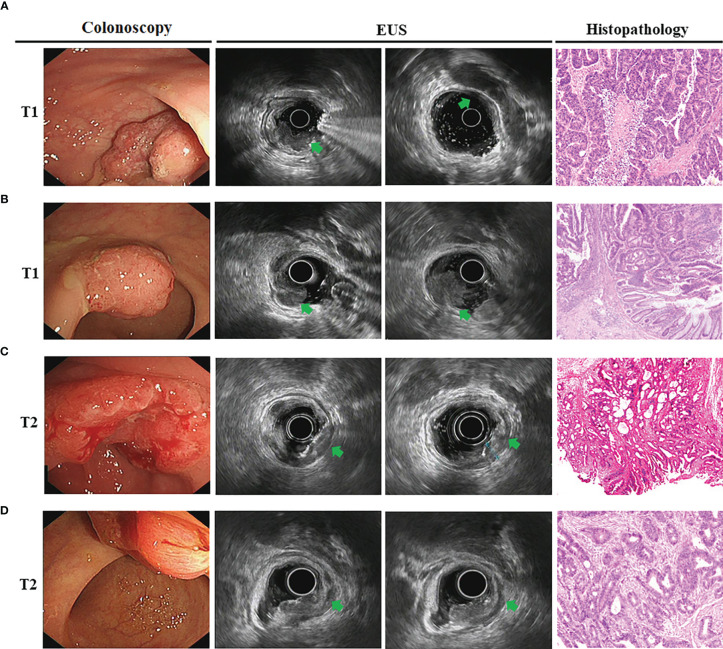
The EUS image features for T1 and T2 tumor T stage. **(A, B)**. Endoscopic view of superficial rectal cancers. Endoscopic images showed the T1 stage lesions infiltrate the mucosa and muscularis mucosae, with submucosa intact (*arrowheads*). Surgical resection confirmed moderately-differentiated adenocarcinoma confined to submucosal layer for male **(A)** and female **(B)**; **(C, D)**. Gastroscopy showed neoplasms located at the rectal walls. EUS images showed disappearance of the first three layers and companied by muscularis propria obvious thickening (*arrowheads*). The surgical specimen confirmed moderately-differentiated adenocarcinoma infiltrated to the muscolaris propria for male **(C)** and female **(D)**.

**Figure 3:**
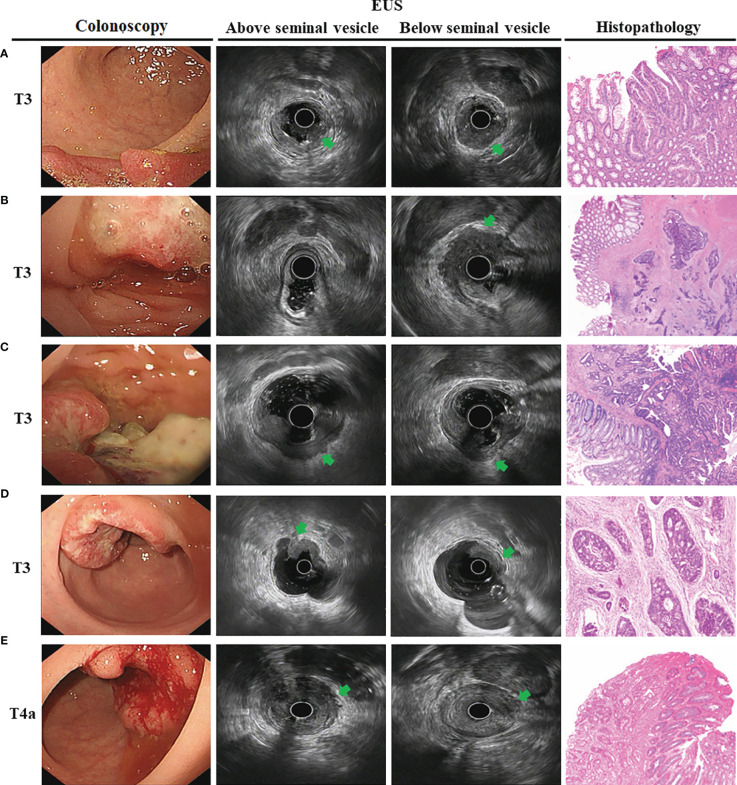
The endoscopic ultrasonography image features in T3 and T4a tumor T stage for male. Endoscopic images of the lesions showed neoplasms located at the rectum with dirty surface. **(A)** EUS images showed a thick hypoechoic lesion spreading from the mucosa to the whole rectal wall. The lesion located at posterior rectum and below the seminal vesicles (*arrowheads*); **(B)** The lesion located at anterior rectum and below the seminal vesicles (*arrowheads*); **(C)** The lesion located at posterior rectum but above the seminal vesicles (*arrowheads*). This T3 tumor penetrates the rectal wall and invaded perirectal fat; **(D)** The lesion located at anterior rectum and above the seminal vesicles. However, hypoechoic lesion invaded to entire wall with an intact serosa layer (*arrowheads*), meaning that the tumor is still limited to the rectal wall. The surgical specimen confirmed tumor confined to the subserosa; **(E)** The lesion located at anterior rectum and above the seminal vesicles. However, hypoechoic lesion invaded to entire wall with irregular rectal wall outer edge (*arrowheads*), meaning that the lesion invaded the rectal serosa. The surgical specimen confirmed tumor infiltrated to the serosa.

**Figure 4:**
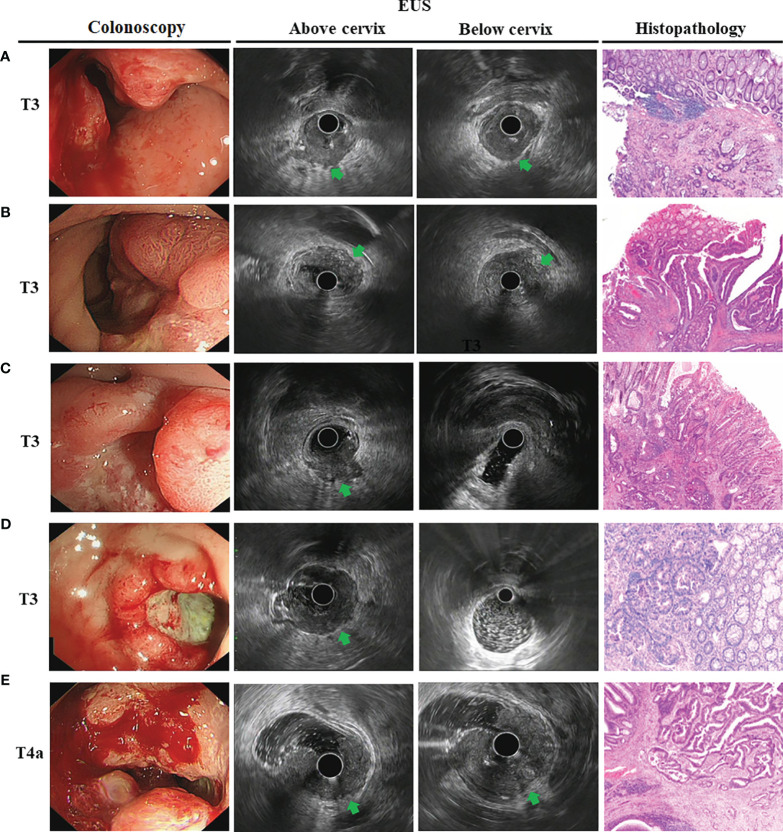
The endoscopic ultrasonography image features in T3 and T4a tumor T stage for female. Endoscopic image showed a large ulcer located the rectal wall covering with moss. **(A)**. EUS image showed an obviously thick hypoechoic lesion that spread throughout the entire wall. The lesion located at posterior rectum and below the cervix (*arrowheads*); **(B)** The lesion located at anterior rectum and below the cervix (*arrowheads*); **(C)** The lesion located at posterior rectum but above the cervix (*arrowheads*). This T3 tumor penetrates the rectal wall and invaded perirectal fat; **(D)** The lesion located at anterior rectum and above the seminal vesicles. However, hypoechoic lesion invaded to entire wall with an intact serosa layer (*arrowheads*), meaning that the tumor is still limited to the rectal wall. The surgical specimen confirmed tumor confined to the subserosa; **(E)** The lesion located at anterior rectum and above the seminal vesicles. However, hypoechoic lesion invaded to entire wall and serosal layer was irregularities in the outer edge of the rectal wall (*arrowheads*), meaning that the tumor had spread outside the serosa. The surgical specimen confirmed lesion infiltrated to the serosal layer.

We then analyzed EUS image features and tried to summarize the characteristics for the different tumor T stages. The hypoechoic change of the first three layers (the mucosal layer to the submucosal layer) was a feature for T1 stage (**Figures 2A, B**). If accompanied with muscularis propria (MP) being indistinctly visible or having obvious thickening was considered to be an indicator that the lesion involved to the MP (T2 stage) (**Figures 2C, D**). The consistency rate between EUS and pathological results for this T2 stage feature was only 67.46% but it had a high positive predictive value (PPV) of 80.05%. Furthermore, when MP disappeared completely and is accompanied with an intact serosal layer was a marker that the lesion involved to the subserosa (T3 stage) (visceral peritoneum covering) (**Figures 3D**, **4D**). Finally, we also found that the irregularities in the outer edge of the rectal wall were markers of rectal serosal layer penetration or arriving at colorectal fat tissue (no visceral peritoneum covering) (**Figures 3**, **4**). The positive predictive value (PPV) for this characteristic is 88.79%. Rectal wall outer edge irregularity is an effective indicator for confirming serosal extension. **Figures 2**–**4** depicted the EUS features of each T stage.

### Seminal Vesicles and Cervix Could be Well Markers for Distinguishing T3 FromT4a Stage

In order to accurately distinguish T3 fromT4a stage, we first divided the tumor site into three segments: upper third from 12 to 15 cm (115 patients, 23.09%), middle third from 8 to 12 cm (309 patients, 62.05%), and lower third from 7 cm to the anus (74 patients, 14.86%) (**Table 2**). Interestingly, using this judgment method, EUS had the lowest accuracy for middle-third tumors (53.07%) but the highest accuracy for lower-third tumors (71.62%) (**Table 4**). The overall accuracy of EUS in classifying rectal T3 and T4a category were 66.04 and 60.09%, respectively. Accuracy results for the T3 stage were 63.48% in the upper third, 53.07% in the middle third, and 71.62% in the lower third rectum. For the T4a stage, 69.93% in the upper third, 52.28% in the middle third, and no T4a stage lesions were located at the lower third rectum. There were statistically significant differences in the EUS accuracy among the different tumor locations (p = 0.029 for T3 stage and p = 0.011 for T4a stage). The middle third rectum is an important anatomical level of peritoneal reflection. This implies that peritoneal reflection is crucial to for distinguishing T3 fromT4a stage.

**Table 4:** Factors affecting EUS T staging accuracy, overstaged and understaged according to clinicopathologic and endoscopic variables by univariate logistic regression analysis.

Variables	No. of accuracy (%)	*P*	No. of overstaged (%)	*P*	No. of understaged (%)	*P*
Cross-sectional portions		0.204		0.583		0.941
Circumferential lesions ≥1/2	201/265 (75.85%)		35/265 (13.21%)		29/265 (10.94%)	
Circumferential lesions <1/2	258/373 (69.17%)		65/373 (17.43%)		50/373 (13.40%)	
		**0.041**		0.883		0.847
Ascites	24/30 (80.00%)		3/30 (10.00%)		3/30 (10.00%)	
Absence of ascites	422/608 (69.41%)		100/608 (16.45%)		86/608 (14.14%)	
		**0.023**		0.305		0.416
MP thickening	73/89 (82.02%)		9/89 (10.11%)		7/89 (7.87%)	
Absence of MP thickening	3368/549 (67.03%)		1111/549 (20.22%)		770/549 (12.75%)	
		**00.009**		00.224		00.774
Rectal wall outer edge irregularity	3310/402 (77.11%)		550/402 (12.44%)		442/402 (10.45%)	
Absence of Rectal wall outer edge irregularity	1155/236 (65.68%)		447/236 (19.92%)		334/236 (14.40%)	
Tumor located at rectum		**00.001**		**00.002**		00.190
Upper third	773/115 (63.48%)		116/115 (13.91%)		226/115 (22.61%)	
Middle third	164/309 (53.07%)		88/309 (28.48%)		57/309 (18.45%)	
Lower third	53/74 (71.62%)		13/74 (17.57%)		8/74 (10.81%)	
Histological type		**0.001**		0.493		**0.001**
Well-differentiated	52/64 (81.25%)		7/64 (10.94%)		5/64 (7.81%)	
Moderately differentiated	305/405 (75.31%)		57/405 (14.07%)		43/405 (10.62%)	
Poorly differentiated	83/120 (69.17%)		21/120 (17.50%)		16/120 (13.33%)	
Signet ring cell adenocarcinoma	29/49 (59.18%)		6/49 (12.25%)		14/49 (28.57%)	

EUS, endoscopic ultrasonography; MP, muscularis propria. The results depend on the AJCC 7th/8th edition. The decimal point is accurate to three digits.

Bold values indicate that P ≤ 0.05 were statistically significant.

Therefore, for further analysis, we then take seminal vesicles and cervix into account as if they are considered as the same anatomical transverse view of peritoneal reflection (**Figures 3**, **4**). Using this new judgment method, the accuracy of EUS for T3 staging increased from 66.04 to 74.26% and T4a staging from 60.09 to 78.45%, respectively. There was a statistically significant difference in EUS accuracy between these two different evaluation criteria (p = 0.006). Collectively, the seminal vesicle or cervix is the important marker to better predict infiltration depth in uT3/T4a rectal cancer.

### Factors Influencing Evaluation of EUS Colorectal Cancer Staging

Univariate and multivariate logistic regression models are generated to test potential associations between lesion characteristics and EUS accuracy. EUS diagnostic accuracy was not influenced by the age, gender, cancer diameter, and circumferential lesions (p >0.05). Compared with patients staged with rectum, those who were staged with colon had more accuracy (71.89% vs. 77.14%, **Table 3**, p = 0.04). Interestingly, univariate logistic regression analysis showed that ascites, MP thickening, rectal wall outer edge irregularity, and lower third tumor location were associated with higher accuracy of EUS (p <0.05, **Table 4**). Rectal wall outer edge irregularity was a significant factor for accuracy by further multivariate logistic regression analysis (p = 0.003; **Table 5**). The middle-third tumors seem to present a significant overstaging (p = 0.002). Further multivariate logistic regression analysis indicated the lesions located at middle-third presented a significantly higher risk of overstaging (p = 0.028, OR = 3.736; **Table 5**).

**Table 5:** Multivariate analysis of clinicopathologic and endoscopic factors affecting EUS T staging.

Variables	*P*	Odds ratio (95% CI)
**Accuracy**		
Rectal wall outer edge irregularity	0.003	3.779 (1.105–8.311)
Middle third rectum	0.012	0.492 (0.090–0.862)
Well-differentiated	0.019	2.723 (1.522–6.198)
Signet ring cell adenocarcinoma	0.001	0.208 (0.049–0.939)
Seminal vesicles and cervix for distinguishing T3 fromT4a stage	0.001	6.859 (2.190–10.865)
**Overstaged**		
Middle third rectum	0.028	3.736 (1.290–6.314)
**Understaged**		
Signet ring cell adenocarcinoma	0.015	4.012 (1.302–9.724)

All variables were calculated by binary or multivariate logistic regression analysis. Results for variables with P >0.05 were not shown. CI, confidence interval.

There were also significant differences in the accuracy among each histological type (p = 0.001). EUS had the highest accuracy for well-differentiated and tended to decline in lesions differentiation getting worse. For well-differentiated tumors, EUS had better staging success relative to that for signet ring cell carcinoma (81.25% vs. 59.18%). The signet ring cell carcinoma had a greater possibility of understaging (p = 0.015, OR = 4.012); More importantly, seminal vesicles or cervix is a good marker for distinguishing T3 fromT4a stage, which is a significant factor for accuracy by univariate logistic regression analysis. When further subjected to multivariate analysis, seminal vesicles or cervix also presented a crucial factor for accuracy for distinguishing T3 fromT4a stage with a 6.859-fold Odds Ratio (p = 0.001) (**Table 5**).

## Discussion

EUS utility to stage colorectal cancer has been recently debated because of reports quoting worse results than those previously published, ranging from 63 to 96% (18–21). The current study describes the EUS T stage accuracy by using two different judgment methods and paying attention to distinguish T3 from T4a T stage. The key findings of this paper are the following: (1) The relationship between the lesions and the seminal vesicles or cervix visualized by EUS might be a predictive factor for distinguishing T3 fromT4a stage; (2) When the lesions are located above the seminal vesicles or cervix, there is a difference between the anterior (T4a) and posterior (T3) walls of the rectum; (3) Rectal wall outer edge irregularity, the tumor location, and histological type were associated with accuracy; (4) The EUS image features of each tumor T stage could guide judgment for EUS gastroenterologist.

A multicenter, prospective, country-wide quality-assurance study at more than 300 hospitals, showed that the pooled uT–pT correspondence of rectal cancer was 64.7% for the EUS of 29,206 patients in Germany (18). Currently, our finding indicates the overall accuracy of EUS in classifying colorectal T category were 73.04%. The results included that colon cancer may be the reason for higher accuracy than reported. For tumor location, the impact on the endosonographic assessment of wall invasion is not settled yet. Some authors reported better accuracy rates for high compared to low rectal tumors (15, 16). There is a significantly better result for tumors within 12 cm of the anal verge. In their opinions, less accurate staging in the lower rectum may have difficulty in reaching all sites of the ampulla recti with a rigid probe. The typical endosonographic five-layer structure of the rectal wall is somewhat less well defined at the level just above the anal canal.

However, there are also contradictory findings (22, 23). Our data would support the latter, in the present study, where inaccuracy was almost completely confined to high and middle rectal tumors. The one reason for the less accurate staging may be a technical shortcoming. It difficult for effacement of the transducer to the tumor when the rigid probe is bent over a colonic bend or in strictures (24). The application of curved radial array echoendoscopes has been limited to the rectum and distal sigmoid colon because of the oblique viewing optics. Colon cancer staging with EUS was not possible until the development of EUS forward viewing radial echoendoscope. It is able to feasibly and safely reach all colonic lesions and within time frames similar to standard colonoscopy procedures and could overcome these limitations (17, 25). The other reason is distinguishing between subserosal and serosal lesions by EUS is indeed challenging. EUS fails to detect peritoneal reflection and ligaments from new AJCC rectal tumor staging version.

So, to date, no group has analyzed the accuracy value of transrectal ultrasound with respect to tumor position on the peritoneal reflection. We used the new method and made sure whether seminal vesicle or cervix as a marker had any influence on the reliability of tumor staging. Analysis showed that there was a significantly difference on the position relationship between the lesions and the seminal vesicles or cervix if they are within reach of the scanner. The impact of endosonographic seminal vesicles or cervix as important instruments is to better predict infiltration depth for distinguishing T3 fromT4a stage. When the lesions invaded throughout the entire wall and are located below the seminal vesicles or cervix, we consider the lesions as T3 stage. If the lesions are located above clearly-defined space between the anterior rectal wall and the posterior surface of the seminal vesicles or cervix, we identify them as T4a stage. If the cancer lesions are located at the posterior rectal wall and above seminal vesicles or cervix, we still consider the lesions as T3 stage.

Furthermore, we also identify factors that affect the accuracy of EUS T staging and found MP disappeared completely and accompanied with an intact serosal layer might be a marker that the lesion involved to the subserosa. The consistency rate was nearly 71%. For serosa invasion, colorectal wall outer edge irregularity is a good indicator of cancer invasion. In addition, the location of the tumor and its histological type are associated with accuracy of EUS staging. Tumor located in middle was an independent indicator that was associated with EUS overstaged and tumors in signet ring cell adenocarcinoma type were related with EUS understaged. The reason may be that tumors differentiate into signet ring cell adenocarcinoma are commonly scirrhous and infiltrative and tend to have tumor microinvasion (26). Microscopic neoplastic invasion into the next layer is undetectable by EUS. These results suggested that careful attention is required during EUS examination and must precede therapeutic schedule for colorectal cancer with these characteristics.

Certainly, the present study has its inherent limitations that require further discussion. First, the sample of patients is relatively small suggesting restricted application of the results; Secondly, T stage with including a subgroup, such as T1a vs. T1b, T4a vs. T4b, could be a further discussion. Finally, the EUS accuracies for N/M staging being not compared is another limitation that should be considered in this study. A multicenter prospective study with a larger patient cohort is required. More data from other centers are warranted to test our results.

In conclusion, EUS could serve as an accurate technology to determine the invasion depth of colorectal cancer. It is worth noting that in this study, the seminal vesicles or cervix should be used to warrant attention while discriminatingly scanning between T3 and T4a disease. Colorectal cancers with location and histological type were more frequently associated with incorrect staging. For these patients, it is recommended that gastroenterologists should consider the T stage image characteristics we mentioned above.

## Data Availability Statement

The original contributions presented in the study are included in the article/supplementary material. Further inquiries can be directed to the corresponding authors.

## Ethics Statement

The studies involving human participants were reviewed and approved by the Ethics Committee of Tongji Medical College, Huazhong University of Science and Technology (IORG No: IORG0003571). The patients/participants provided their written informed consent to participate in this study.

## Author Contributions

CH contributed to study concept and design, analysis and interpretation of data, drafting of the manuscript. TX and KZ contributed to statistical analysis. MY contributed to the pathology results. JL contributed to the acquisition of data and research performance. ZD and RL designed, supervised the study and revised the manuscript as the corresponding author. All authors contributed to the article and approved the submitted version.

## Funding

This study was supported in part by the National Natural Science Foundation of China (Nos. 81800467, 81720108006, 81330014, 81770637, and 82170570).

## Conflict of Interest

The authors declare that the research was conducted in the absence of any commercial or financial relationships that could be construed as a potential conflict of interest.

## Publisher’s Note

All claims expressed in this article are solely those of the authors and do not necessarily represent those of their affiliated organizations, or those of the publisher, the editors and the reviewers. Any product that may be evaluated in this article, or claim that may be made by its manufacturer, is not guaranteed or endorsed by the publisher.
